# Automated segmentation by deep learning of loose connective tissue fibers to define safe dissection planes in robot-assisted gastrectomy

**DOI:** 10.1038/s41598-021-00557-3

**Published:** 2021-10-27

**Authors:** Yuta Kumazu, Nao Kobayashi, Naoki Kitamura, Elleuch Rayan, Paul Neculoiu, Toshihiro Misumi, Yudai Hojo, Tatsuro Nakamura, Tsutomu Kumamoto, Yasunori Kurahashi, Yoshinori Ishida, Munetaka Masuda, Hisashi Shinohara

**Affiliations:** 1 Department of Surgery, Yokohama City University, Kanagawa, Japan; 2 Anaut Inc., Tokyo, Japan; 3 Incubit Inc., Tokyo, Japan; 4 Department of Biostatistics, Yokohama City University School of Medicine, Kanagawa, Japan; 5 Department of Gastroenterological Surgery, Hyogo College of Medicine, 1-1 Mukogawa-cho, Nishinomiya, Hyogo 663-8501 Japan

**Keywords:** Cancer, Anatomy, Gastroenterology

## Abstract

The prediction of anatomical structures within the surgical field by artificial intelligence (AI) is expected to support surgeons’ experience and cognitive skills. We aimed to develop a deep-learning model to automatically segment loose connective tissue fibers (LCTFs) that define a safe dissection plane. The annotation was performed on video frames capturing a robot-assisted gastrectomy performed by trained surgeons. A deep-learning model based on U-net was developed to output segmentation results. Twenty randomly sampled frames were provided to evaluate model performance by comparing Recall and F1/Dice scores with a ground truth and with a two-item questionnaire on sensitivity and misrecognition that was completed by 20 surgeons. The model produced high Recall scores (mean 0.606, maximum 0.861). Mean F1/Dice scores reached 0.549 (range 0.335–0.691), showing acceptable spatial overlap of the objects. Surgeon evaluators gave a mean sensitivity score of 3.52 (with 88.0% assigning the highest score of 4; range 2.45–3.95). The mean misrecognition score was a low 0.14 (range 0–0.7), indicating very few acknowledged over-detection failures. Thus, AI can be trained to predict fine, difficult-to-discern anatomical structures at a level convincing to expert surgeons. This technology may help reduce adverse events by determining safe dissection planes.

## Introduction

Technological innovations in optics and robotics help to support and improve the surgeon’s eyes and hands, and yet adverse surgical events remain an unsolved problem^1–3^. According to a report on human performance errors during surgery, nearly 30% of surgical complications are caused by misrecognition (MR) during operations^4^. Fatigue from long shifts or prolonged surgeries reduces surgeons’ cognitive ability and performance^5^, and they can also experience fluctuations in cognition and attention based on their physical and mental condition. Moreover, inexperienced surgeons tend to have insufficient anatomical knowledge and techniques for managing intraoperative events such as bleeding^6^. Further technological innovations that can support the “surgeon's brain” may help to improve surgical outcomes.

Performing surgery is not unlike driving a vehicle, in that decisions and maneuvers must be made based on visual information. In recent years, the technologies behind autonomous driving systems that utilize artificial intelligence (AI) technology, especially deep-learning algorithms, have progressed rapidly^7^. One benefit that AI offers in that field is fewer traffic accidents due to human error, by predicting safe driving lanes based on obstacle recognition (e.g., of other vehicles, traffic lights, road signs, and pedestrians). For example, the Japanese automobile manufacturer Subaru has reported that its driver assistance system has reduced car accidents by 60%^8^. Applying similar technologies to surgery could be a way to support surgeons’ experience and skills, mitigating fluctuations in cognition and attention due to their physical and mental condition while operating^9^.

To continue the analogy, in gastrointestinal cancer surgery the “driving lane” is the dissection plane, referred to as the “Holy Plane” in total mesorectal excision^10^ and considered to be a common anatomy in colonic^11,12^, esophageal^13,14^, and gastric surgery^15,16^. The dissection plane is an avascular space consisting of loose connective tissue fibers (LCTFs) that appears when expanded by optimal countertraction^12,15,16^. Accumulating evidence has revealed that a sharp dissection of LCTFs not only improves oncological outcomes, but also reduces surgical complications^17–19^. In this study, we explored the use of deep learning in medical image analysis to identify this complex and difficult-to-discern anatomy within the surgical field. We aimed to develop an AI model that achieves LCTF predictions which are highly convincing to expert surgeons and help surgeons visualize safe dissection planes during lymphadenectomy in robot-assisted gastrectomy.

## Methods

### Video dataset

Videos of robot-assisted surgeries for gastric cancer performed at the Hyogo College of Medicine, Japan, from May 2018 to January 2020 were used to develop and evaluate the AI algorithm. These operations were performed using the da Vinci Xi Surgical System (Intuitive Surgical, Sunnyvale, CA) by board-certified surgeons (H.S., Y.I., Y.K., and T.K.) who were certified as Console Surgeons through da Vinci Surgical System Off-site Training. The recording system (AVCCAM AG-MDR15, Panasonic, Osaka, Japan) produced videos with framerates of 30 frames per second (fps). We selected videos that captured suprapancreatic lymph node dissections, because this operative step is not complicated, is well formalized, and the dissection plane is easily visualized. The 33 eligible videos were clipped and downloaded to a hard drive. The videos were then categorized according to use: 20 for training the algorithm, 3 for validation, and 10 for evaluation.

### Annotation and deep learning

**Figure 1:**
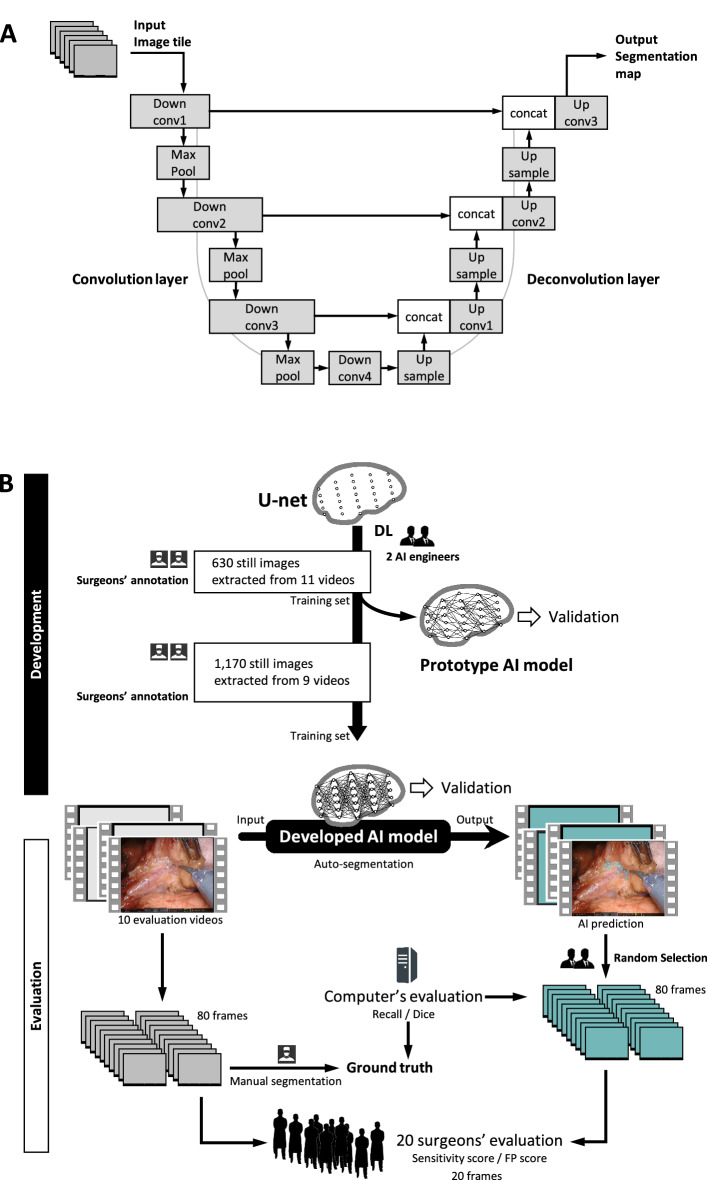
Deep learning algorithm and the AI model developed in this study. (**a**) The deep-learning architecture implementing U-Net. Conv, convolution; concat, concatenation. (**b**) Development and performance evaluation of the AI model. *MR* misrecognition.

Still images, including at least 10 with clearly depicted LCTF structures, were framed from the training videos and saved in BMP format at a resolution of 1920 × 1080 pixels (aspect ratio 16:9). To create the training set, the boundaries of each LCTF were precisely annotated on each frame by two surgeons (N.K. and Y.K.) who have completed a fellowship in gastroenterological surgery and have experience performing more than 100 laparoscopic gastrectomies. The neural network model was based on the convolutional neural network U-net architecture, which has previously shown promising results in segmentation tasks, particularly for medical images^20–22^. Figure 1a shows our deep learning algorithm, which allows more accurate output of segmentation maps by extracting object features in the convolution layer while restoring positional information in the deconvolution layer. Model training and inference were performed on a workstation with a Tesla V100 GPU (NVIDIA Corp., Santa Clara, CA) with 32 GB memory. The LCTF detection threshold was set to 50%. Automated segmentation results were output at around 5 fps by highlighting the LCTF area in turquoise.

### Development of the AI model

A prototype AI model was produced in May 2019 using 630 images taken from 11 of the training videos. As Fig. 1b shows, the U-net deep learning algorithm was developed by augmenting the training data with surgeons’ annotations. The process of developing the prototype AI model to the latest one was carried out through more sophisticated annotations and data augmentation without changing the architecture of U-net. Performance of the developed AI model was carefully verified using the 3 validation videos, separate from the training ones. The latest AI model was trained using a total of 1800 images, including more than 20,000 LCTF annotations taken from the 20 training videos.

### Model evaluation by computation

Three engineers (E.R., P.N., and N.K.) randomly sampled 80 frames from the 10 evaluation videos that underwent LCTF prediction using the latest AI model (see Fig. 1b). Two annotators (N.K. and Y.K.) manually segmented the corresponding frames from the original to create the ground truth. Agreement was quantitatively evaluated by measuring spatial overlap of the number of pixels between the actual area concordant with surgeons’ manual segmentations (i.e., the ground truth) and the predicted area of the AI’s automated segmentation, using Recall^23^ and F1/Dice^24,25^ scores. These are the most commonly used performance metrics in machine learning for assessing sensitivity and similarity, respectively, calculated as

$$\mathrm{Recall}=\frac{\mathrm{TP}}{\mathrm{TP}+\mathrm{FN}}$$

$$\frac{\mathrm{F}1}{\mathrm{Dice}}=\frac{\mathrm{TP}}{\mathrm{TP}+\frac{1}{2}(\mathrm{FP}+\mathrm{FN})}$$

where TP, FN, and FP respectively represent true positive, false negative, and false positive counts.

### Model evaluation by trained surgeons

**Figure 2:**
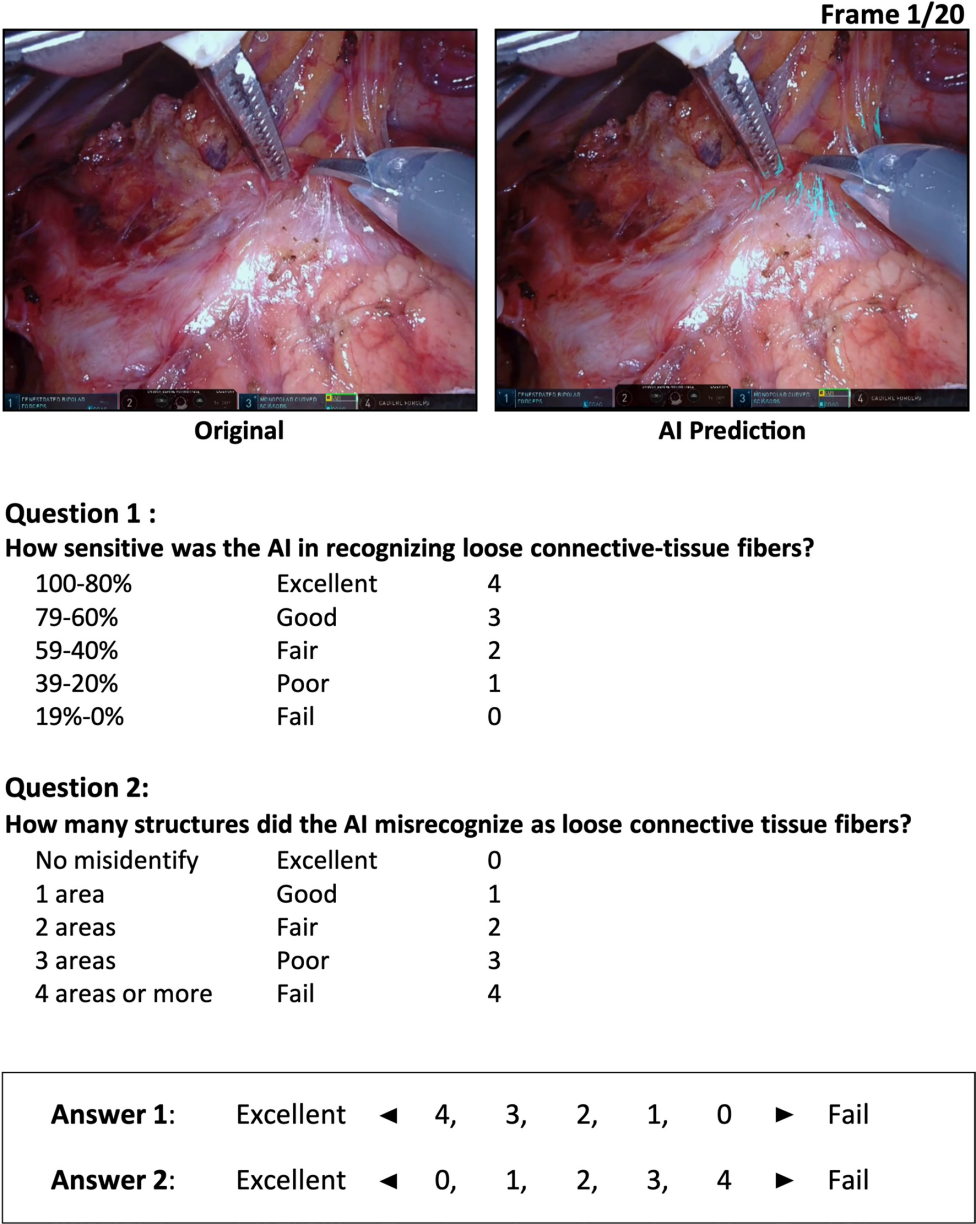
The questionnaire for qualitative evaluation of the AI’s segmentation performance completed by expert surgeons.

In quantitative evaluations such as the F1/Dice score and Recall, it is difficult for clinician to interpret values to judge validity for clinical application, especially in cases of evaluations related to visual or cognitive performance. Therefore, we created a questionnaire with reference to previous studies for the purpose of complementing the quantitative evaluation^26–28^. Model performance for 20 of the 80 test frames was also evaluated qualitatively by a two-item questionnaire completed by 20 trained gastrointestinal surgeons (Fig. 1b). Each test frame was sequentially projected onto a high-resolution screen alongside the original frame (Fig. 2), and the evaluators intuitively answered the questionnaire. The first question was: *Q1. How sensitive was the AI in recognizing loose connective tissue fibers?* The answers were scored for recognition on a 5-point scale at 20% increment (0 for lowest recognition [0%–19%] to 4 for highest recognition [80%–100%]). The mean score for each frame was used as the sensitivity score. The second question was: *Q2. How many structures did the AI misrecognize as loose connective tissue fibers?* These answers were also scored on a 5-point scale (0 for no MR areas to 4 for 4 or more MR areas). The mean score for each frame was used as the MR score.

### Statistical analysis

The sensitivity and MR scores were plotted as a scatter diagram and a confidence ellipse with probability 0.95 was drawn. The correlation between Recall and sensitivity scores was assessed by calculating the Pearson correlation coefficient. JMP Pro version 15 software (SAS Institute Inc., Cary, NC) was used for statistical analysis.

### Ethics approval and consent to participate

This study was approved by the Ethics Committee of the Hyogo College of Medicine (Approval number 3057). All procedures performed in studies involving human participants were in accordance with the ethical standards of the institutional and/or national research committee and with the 1964 Helsinki declaration and its later amendments or comparable ethical standards. All participants provided informed consent to video recording of their cases before surgery in the study, and data were completely anonymized.

## Results

**Figure 3:**
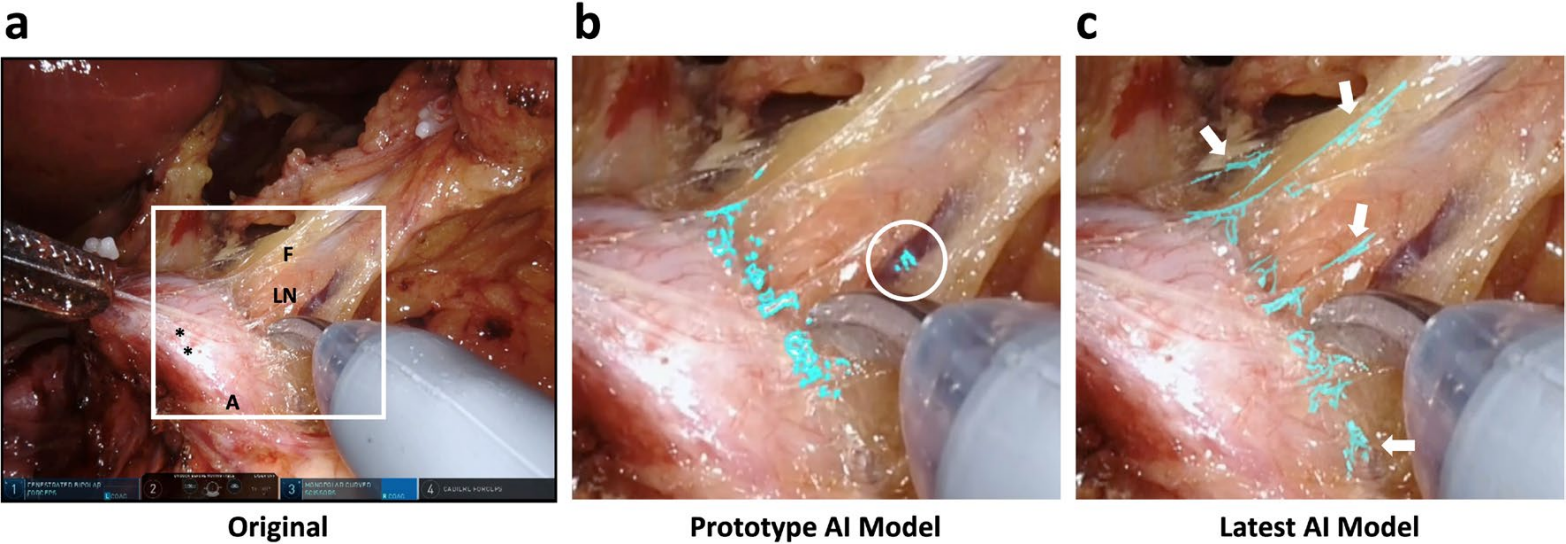
Comparison of segmentation performance at different stages in deep learning. (**a**) An original frame. *CHA* common hepatic artery, *F* fat tissue, *LN* lymph node; *, nerve. (**b**) Magnified view of the square in **A** showing prediction of loose connective-tissue fibers (LCTFs) highlighted in turquoise by the prototype AI model. White circle indicates an area of over-detection. (**c**) Prediction by the latest AI model. Arrows indicate LCTFs that could not be detected by the prototype AI model.

Figure 3 shows the results of automated segmentation at different stages in deep learning using the same frame in a validation video. In the prototype model, the AI had already learned to approximately highlight LCTF features from a vast number of image pixels representing other anatomical landmarks (e.g., arteries, lymph nodes, and fat tissue) and surgical instruments. It also discriminated LCTFs from nerves with similar fine, white features. However, when magnified (Fig. 3b), the outline was still ambiguous and there were some undetected or over-detected areas. With more sophisticated annotations and data augmentations, the latest model segmented the LCTFs more sharply and naturally, and recognition failures were significantly reduced (Fig. 3c).

The Electronic Supplementary Material (Video 1) shows examples of outputs from the latest AI model compared with those from the original. The AI accurately highlighted the LCTFs as soon as the dissection plane appeared due to the surgeon’s countertraction. Note that this segmentation was done on the operative video retrospectively, although it seems in the video that the surgeon is cutting while confirming LCTFs highlighted by the AI.

The mean Recall and F1/Dice scores of the 80 test frames were 0.605 (range 0.230–0.909) and 0.525 (range 0.263–0.712), respectively, showing acceptable sensitivity and similarity between the automated and manual segmentations. Of these 80 test frames, 20 frames were used for the qualitative evaluation. Table 1 summarizes the performance metrics measured by computation and qualitative scores assigned by the evaluators for each of the 20 frames. The mean Recall score (0.606, range 0.230–0.861) and mean F1/Dice score (0.549, range 0.335–0.691) were comparable to the results for the 80 test frames. In the qualitative evaluation by surgeons, the mean sensitivity score was 3.52 (range 2.45–3.95). Note that 88.0% of evaluations were the highest score of 4, indicating that the evaluators were generally convinced by the LCTF segmentation output from the AI. Furthermore, the mean MR score was a low 0.14 (range 0–0.7), indicating very few acknowledged MR failures.

**Table 1:** Performance metrics and qualitative scores in the 20 randomly sampled video frames.

Frame	Recall score	F1/Dice score	Sensitivity score mean (SD)	MR score mean (SD)
1	0.792	0.532	3.80 (0.41)	0.05 (0.22)
2	0.522	0.509	3.40 (0.50)	0.20 (0.41)
3	0.583	0.587	3.90 (0.31)	0.55 (0.60)
4	0.338	0.341	3.75 (0.44)	0.70 (0.57)
5	0.626	0.630	3.35 (0.75)	0
6	0.750	0.642	3.90 (0.31)	0
7	0.445	0.571	3.20 (0.70)	0.20 (0.41)
8	0.609	0.462	3.90 (0.70)	0
9	0.819	0.601	3.90 (0.70)	0
10	0.861	0.587	3.95 (0.22)	0.10 (0.31)
11	0.230	0.335	2.50 (0.61)	0
12	0.458	0.521	2.95 (0.76)	0
13	0.777	0.691	3.80 (0.41)	0
14	0.667	0.649	3.55 (0.60)	0.35 (0.59)
15	0.544	0.511	3.25 (0.55)	0.05 (0.22)
16	0.748	0.621	3.65 (0.59)	0
17	0.660	0.575	3.75 (0.55)	0.05 (0.22)
18	0.705	0.590	3.95 (0.22)	0.45 (0.51)
19	0.454	0.493	2.45 (0.60)	0.05 (0.22)
20	0.538	0.541	3.40 (0.60)	0
Mean	0.606	0.549	3.52 (0.46)	0.14 (0.21)

*MR* misrecognition, *SD* standard deviation.

**Figure 4:**
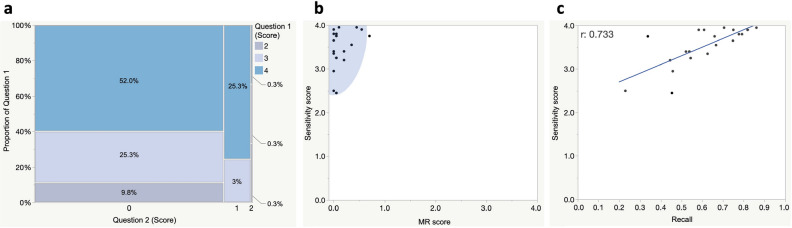
Relations between computed performance metrics and qualitative scores. (**a**) A mosaic diagram showing the distribution of all scores assigned by 20 evaluators to 20 randomly sampled frames. Blue, light blue, and gray panels respectively represent scores of 4, 3, and 2 for Question 1 (see Fig. 2). Vertical and horizontal axes respectively represent the proportion of scores assigned to Questions 1 and 2. Values in the rectangles represent the ratio of each category against the total. There were no scores below 1 for Question 1 and no scores above 3 for Question 2. (**b**) Scatter plot showing the relation between sensitivity and misrecognition (MR) scores for each frame. Blue area is the confidence ellipse, representing the area of 95% probability that the plots exist. (**c**) Scatter plot showing the relation between sensitivity and Recall scores. The correlation coefficient was 0.733 and the 95% confidence interval was 0.430–0.887. Blue line represents the regression formula, calculated as Y = 2.302 + 2.001X. *Y* sensitivity score, *X* Recall score.

We further analyzed the relation between the performance metrics and qualitative scores. Figure 4a shows a mosaic diagram showing the distribution of all scores for each question assigned by the 20 evaluators to the 20 sampled frames. The most common response (from 52.0% of evaluators) was a score of 4 for Question 1 and 0 for Question 2, followed by a score of 3 for Question 1 and 0 for Question 2 (25.3%). No evaluators scored Question 1 as 1 or less, nor did they assign 3 or more to Question 2. The scatter plot in Fig. 4b shows the relation between sensitivity scores and MR scores for each frame. The sensitivity scores showed some variation in the high range among samples, but the MR scores were generally low, so the 95% confidence ellipse converged on the upper left corner of the coordinates. Figure 4c shows the relation between the sensitivity scores and Recall. A strong correlation with a correlation coefficient of 0.733 (95% CI 0.430–0.887) was revealed between the two sensitivity parameters. The regression equation was Y = 2.302 + 2.001X (Y: Sensitivity score; X: Recall), suggesting that surgeon evaluators are more convinced than the performance metrics.

**Figure 5:**
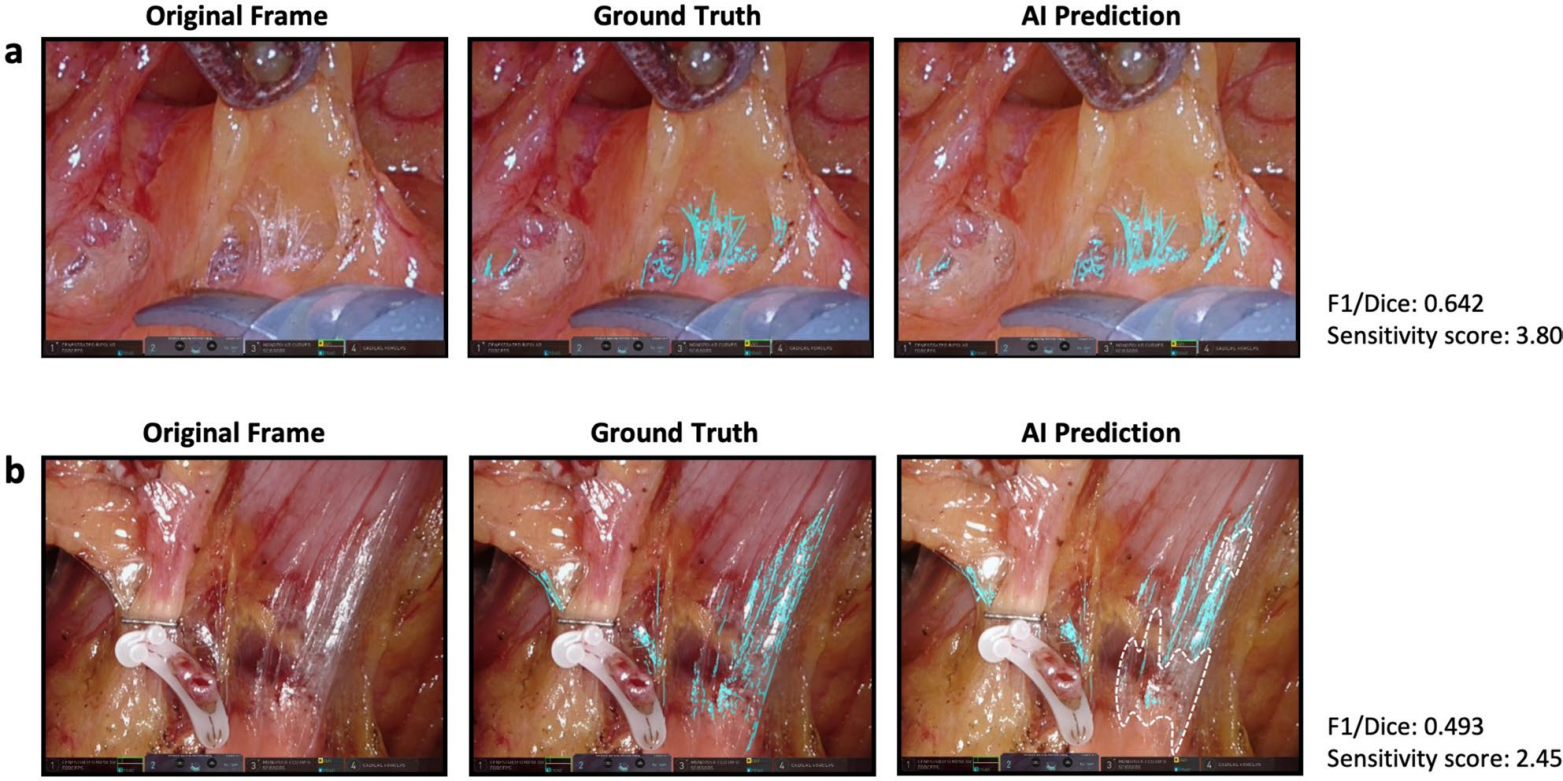
AI prediction results for (**a**) frame 6 with the highest sensitivity score and (**b**) frame 19 with the lowest sensitivity score. The area surrounded by the broken line is an under-detection area.

Figure 5 shows two examples of AI predictions. Based on human judgment, in frame 6 the AI seemingly completely segments the LCTF, in that the results are nearly identical to the manually segmented areas of the ground truth. Indeed, 18 of the 20 surgeons assigned Question 1 the highest score, and the sensitivity score was 3.80. Even so, the F1/Dice score was only 0.642, probably due to overemphasis of slight deviations. In frame 19, there is a clear discrepancy between the AI’s segmentation results and the ground truth. Surgeon evaluations were lowest for this frame, with a sensitivity score of 2.45. The F1/Dice score was also a low 0.493, probably due to under-detection of translucent LCTFs.

**Figure 6:**
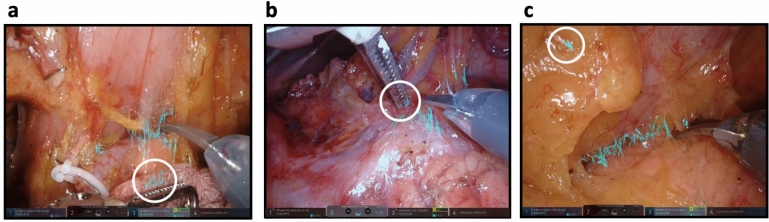
Examples where the AI misrecognized (**a**) gauze mesh fiber, (**b**) fine grooves at the tips of forceps, and (**c**) minor halation of fat or blood surfaces as loose connective tissue.

Nine of the 20 sampled frames had no areas that were judged as false recognitions. There were up to two misrecognitions in each of the remaining 11 frames. Specifically, the AI misrecognized features such as gauze mesh fiber (Fig. 6a), fine grooves at the tips of forceps (Fig. 6b), and minor halation of fat or blood surfaces (Fig. 6c) as LCTFs.

## Discussion

In this study, we demonstrated the feasibility of using AI to automatically segment LCTFs to define safe dissection planes during lymphadenectomy in intraoperative videos of robot-assisted gastrectomy. The method’s performance was quantitatively demonstrated (by mean Recall and F1/Dice scores of 0.605 and 0.525, respectively) and was qualitatively convincing to expert surgeons. Notably, there were nearly no MRs. This study is the first to show that AI developed through deep learning can precisely identify fine surgical anatomy.

AI algorithms, particularly those for deep learning, have advanced considerably in medical image-recognition tasks such as radiography^29–31^, endoscopy^32,33^, and pathological diagnosis^34,35^, but their applications to surgery are still being investigated. Many attempts have aimed to recognize surgical instruments^36^ or operative workflows such as cholecystectomy^37–39^, colectomy^40^, and sleeve gastrectomy^41^. Madani et al. reported promising results for the identification of safe zones for dissection during laparoscopic cholecystectomy (defined as the area located within the hepatocystic triangle), with high sensitivity and F1/Dice scores of 0.69 and 0.70, respectively^37^. In the present study, we assigned AI the more difficult task of recognizing LCTFs for direct visualization of safe dissections planes. The feasible results obtained may be due to augmentation of more than 1800 training data, including over 20,000 objects from intraoperative videos in which surgical fields were stabilized by robotic equipment. In addition to the dataset size, annotation consistency could be especially important when recognizing indefinite regions of interest such as LCTFs, because preciseness of the ground truth greatly affects the outcome of supervised learning. In this study, we used training data carefully labeled by surgeons with clinical experience in gastric cancer surgery. Annotation reliability is indicated by a strong correlation between the Recall scores calculated using surgeons’ annotations as the ground truth and the sensitivity scores assigned by trained surgeon evaluators.

Performance metrics in machine learning highly rely on pixel-wise deviation between the two sets and are biased according to the shape of segment regions^23^. We used F1/Dice scores because they reflect the size and location agreement for object segmentation^42^. However, when compared with human vision, the values for fine structures such as fibers are underestimated, because slight deviations increase FP and FN, which are used in the denominator of the calculating formulas^25^. In this study, the mean F1/Dice score of 0.525 was not necessarily higher than those used by Madani et al.^37^ to identify the liver and gallbladder (0.86 and 0.72, respectively). However, as shown in Fig. 3 and the Supplementary Videos, it is clear that the AI exactly highlights LCTFs without any visual disagreement. Indeed, these subjective impressions are supported by the results shown in Fig. 4, namely that most surgeons were convinced by the AI’s prediction of LCTFs. Considering that the value was only 0.642 even in frame 6, to which 90% of trained surgeons assigned the highest sensitivity score, we believe the F1/Dice score demonstrates acceptable performance. As computer segmentation tasks expand to the field of surgery, it will be necessary to discuss how small deviations beyond human discernment are problematic. Additional research is therefore needed to develop better metrics.

Those LCTFs that the evaluators judged to be inadequately predicted by the AI shared the common characteristics of translucency and blurring. One cause for such under-detection errors is that the detection threshold was set to 50%, but experts empirically know where LCTFs appear in the tissue deployed by countertraction^19^, making it easy to recognize any discrepancy between what is actually seen and the segmentations produced. Interestingly, medical students assigned higher scores in the same questionnaire (data not shown). In other words, expert surgeons require AI to have higher levels of predictive ability than humans with anatomical knowledge but no surgical experience. With further learning, AI will be able to predict operative procedures and display surgical anatomy that can be identified only by highly experienced surgeons. Capabilities for sharing an image of the dissection plane with others will enhance common understanding and facilitate surgery. Further, displays highlighted with a probability heat map will be more useful for probabilistic predictions of safe or dangerous dissection planes. Video-based coaching is known to be an efficient teaching method for surgical residents^43^, our results could be utilized as automated coaching early in surgical education.

The most important application of automated anatomy segmentation is to support surgeons’ decision making. Even with technological advances in surgical optics, the outcome of an operation still ultimately depends on the surgeon’s experience and expertise^6,44^ and cognition due to physical and mental condition^5^ during the operations, so automated image segmentation technology can improve the safety and outcome of surgery by supporting decision making. The six levels of autonomous driving as defined by the Society of Automotive Engineers range from 0 (fully manual) to 5 (fully autonomous), with levels up to 2 classified as “driving assistance” that includes steering correction to maintain a “driving lane”^45^. Recent studies on level-2 driver assistance systems suggest that such technologies reduce driving stress and accidents^46^. Recently, Yang et al. proposed a roadmap toward full automation of surgery^47^, where level 2 is defined as task autonomy in which the robot autonomously performs specific human-designated tasks. Similar to the evolution of automated vehicles, real-time display of AI-analyzed visual data could eventually be incorporated into advanced robotic surgery platforms to help surgeons maintain a safe “dissection plane”.

While our results show promise for clinical use, there are some limitations to consider. First, our AI model has not yet been trained to accurately identify LCTFs under possible intraoperative conditions such as bleeding, which can blur boundaries and change colors. Overcoming this challenge is essential to our goal of developing deep-learning models that improve surgical safety by pairing surgery and AI technologies. Creating training data from surgical videos performed by highly experienced surgeons in difficult situations will improve segmentation performance. Second, we need to evaluate the method’s versatility. Generally, AI models can make inferences and predictions based on the training dataset only. However, LCTFs are common anatomy that appears in the optimal dissection plane in many areas of surgery^10–16^, and we preliminarily confirmed that the algorithm trained using a gastrectomy dataset also segments LCTFs in total mesorectal excision videos. Third, the mean inference framerate of the AI model was only 4.9 fps, so the real-time predictions needed for operating room deployment have not been achieved. However, due to improved machine learning methods, this value is recently approaching 30 fps, so we should soon be ready to bring this model to the operating room.

## Conclusions

Deep-learning algorithms can be trained to predict fine, difficult-to-discern anatomical structures such as LCTFs in intraoperative videos at a level that is convincing to expert surgeons. This technology can be used to assist in real-time decision making by presenting a safe dissection plane, which in turn can reduce adverse events. Newer and more advanced algorithms for image segmentation will become increasingly available in surgical fields to provide higher performance and safety.

## Supplementary Information

Supplementary Video 1.

## Data Availability

We cannot share the data and materials because the Ethics Committee of Hyogo College of Medicine prohibit publication of raw data base including patients’ clinical data even in the case that identifying/confidential data are not included.
